# Attitudes, perceptions, and factors influencing the adoption of artificial intelligence Among healthcare professionals in Saudi Arabia: a UTAUT-based study

**DOI:** 10.3389/frhs.2026.1895739

**Published:** 2026-08-27

**Authors:** Yara Alrashed, Nouf Alsaheil

**Affiliations:** Department of Health Administration, College of Business Administration, King Saud University, Riyadh, Saudi Arabia

**Keywords:** artificial intelligence, digital health, healthcare professionals, Saudi arabia, technology adoption, UTAUT

## Abstract

**Background:**

Amid the global race toward intelligent healthcare systems, Saudi Arabia stands at a pivotal moment in its digital health transformation. Understanding how prepared healthcare professionals are to adopt artificial intelligence is essential for shaping successful national strategies.

**Objectives:**

This study aimed to assess healthcare professionals’ attitudes, perceptions, and intentions toward using AI in clinical practice; examine awareness and actual use; identify key predictors of AI adoption based on the Unified Theory of Acceptance and Use of Technology (UTAUT); and explore the mediating role of institutional support.

**Methods:**

A cross-sectional survey was conducted among 521 healthcare professionals, including physicians, nurses, administrators, and allied health workers, across Saudi Arabia. The survey assessed awareness, usage, perceived usefulness, ease of use, social influence, facilitating conditions, perceived risks, and the intention to use AI. Data were analyzed using chi-squared tests, multiple regression, and mediation analyses.

**Results:**

Although AI awareness was remarkably high (89.1%) and optimism toward the future was strong (79.0%), only 51.2% of participants reported actual clinical use of AI, A 37.9 percentage-point awareness–use gap. Two factors consistently stood out as powerful drivers of intention: believing that AI is genuinely useful (*B* = 0.491, *p* < .001) and feeling confident in one's ability to use it (*B* = 0.224, *p* < .001). Institutional support played an important but mostly indirect role in shaping intentions by enhancing these two beliefs. Social influence had little effect and was negative for nurses, whereas perceived risk did not significantly deter adoption. Despite structural and ethical challenges, intention to use AI remained high (71.6%), A 20.4 percentage-point gap ahead of actual use, indicating that organizational barriers, rather than individual willingness, remain the primary obstacle to AI integration.

**Conclusion:**

These dynamics provide unique opportunities. With 71.6% of professionals intending to adopt AI, targeted training initiatives, clearer governance frameworks, and organizational support may help facilitate the sustainable integration of AI into healthcare practice. This study offers empirical evidence and a roadmap for transforming enthusiasm into sustainable, safe, and meaningful AI integration, and may support healthcare leaders and policymakers in developing strategies for safe and sustainable AI integration within the Saudi healthcare system.

**Practical Implications:**

Advancing AI-enabled healthcare in Saudi Arabia requires investment not only in technology, but also in healthcare professionals’ preparedness and organizational support. Structured training programs, hands-on exposure to AI tools, supportive leadership, adequate infrastructure, and clear data governance frameworks may help healthcare professionals adopt AI more confidently and sustainably within clinical practice.

## 1 Introduction

The Saudi healthcare sector has undergone significant reforms and has adopted advanced technologies such as artificial intelligence (AI) to enhance services in alignment with the Vision 2030 human capital development goals (1). Saudi Vision 2030 is a national reform strategy aiming at diversifying the economy and enhancing the efficiency and quality of public services. In the healthcare industry, it stresses digital transformation, technical innovation, workforce development, and adoption of data-driven healthcare solutions (2, 3). Therefore, it is particularly crucial to understand the intention of healthcare professionals to implement AI, to evaluate the sector's preparedness to reach these strategic objectives. In this context, AI refers to a computerized system capable of executing physical and cognitive tasks, handling complex problems, and making autonomous decisions without direct human guidance (4). As a transformative domain in computer science, AI has been increasingly integrated into healthcare delivery to enhance efficiency, accuracy, and service quality (5). AI can be utilized as a clinical decision support (CDS) system to assist in patient-specific diagnostic and therapeutic decisions, as well as in population-based risk prediction analytics (4). These innovative applications can improve diagnostic accuracy, support better treatment decisions, enhance clinical workflow efficiency, and improve patient outcomes (6). In addition to clinical applications, AI technologies can transform administrative and operational processes among healthcare providers, payers, and pharmaceutical organizations.

Although administrative applications may appear less prominent than direct patient care, they still generate significant operational efficiency. Despite these encouraging developments, the integration of AI technologies into routine clinical practice remains a major challenge. Successful implementation requires regulatory approval, integration with electronic health record systems, product standardization, clinician training, reimbursement mechanisms, and continuous system updates (7). Addressing these interdependent requirements adds considerable organizational and operational complexity to AI adoption.

Against this backdrop, healthcare professionals, including physicians, nurses, allied health staff, and healthcare leaders, are the primary users of AI-based clinical systems and play a central role in determining whether these technologies are successfully integrated into practice. A persistent AI adoption gap is documented across international studies: despite 72.75% of physicians and nurses reporting positive intentions toward medical AI, actual utilization in clinical practice was merely 19.11% in a nationwide survey (8) a 53.6 percentage-point gap between intention and use. Similarly, a cross-sectional study reported that although approximately 70% of healthcare professionals demonstrated awareness and positive perceptions of AI applications, only approximately 27% reported practical exposure or use of AI tools, a gap of roughly 43 points (9). This evidence indicates that favorable perceptions alone are insufficient to translate into routine implementation, and it underscores the critical role of organizational support, workforce competency, and implementation readiness in closing the gap between attitude and behavior.

This gap is further reinforced by additional survey evidence showing that 80.1% of allied health professionals did not use AI in their practice and 87% reported limited knowledge of AI technologies, with workforce knowledge and skill gaps identified as major implementation challenges (10). Consistent with these findings, a recent study reported that although 51.8% of healthcare professionals expressed positive perceptions of AI applications, the majority demonstrated suboptimal knowledge (68.7%) and low levels of practical use (53.4%) (11). Taken together, these international findings consistently indicate that favorable perceptions alone are insufficient to ensure effective AI integration. Instead, workforce competency, digital literacy, institutional support, and facilitating conditions appear to play decisive roles in shaping adoption outcomes. AI implementation, therefore, represents not just technological advancement, but also a complex socio-organizational transformation requiring behavioral and systemic preparedness.

**Figure 1:**
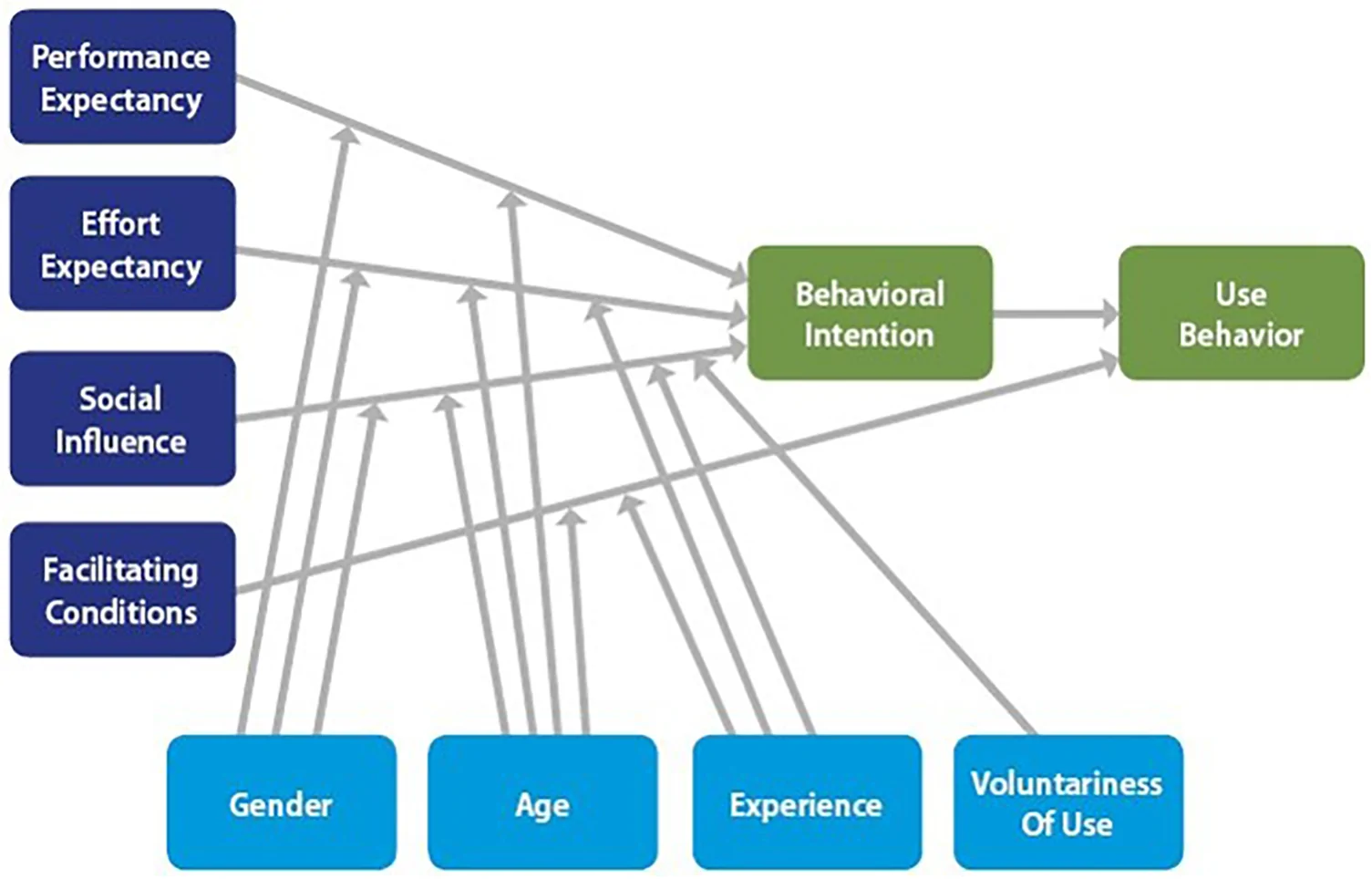
Unified theory of acceptance and Use of technology (UTAUT). Adapted from Venkatesh et al. (12).

The Unified Theory of Acceptance and Use of Technology (UTAUT), developed by Venkatesh et al. (12), is a well-established framework for explaining technology adoption. The model proposes that individuals’ behavioral intention to use a technology is primarily influenced by performance expectancy, effort expectancy, and social influence, whereas actual use is determined by both behavioral intention and facilitating conditions. Performance expectancy is defined as “the degree to which an individual believes that using the technology will help him or her attain gains in job performance,” whereas effort expectancy refers to the perceived ease of use. Social influence captures the extent to which individuals perceive that important others believe they should use the technology, and facilitating conditions refer to the availability of organizational and technical support that enables technology use. The conceptual framework based on the UTAUT model is presented in Figure 1.

In Saudi Arabia, the Ministry of Health actively supports digital infrastructure development and healthcare quality improvement initiatives to advance the national digital health transformation (3). Current evidence suggests that AI adoption in routine clinical practice remains modest, with only 31.6% of healthcare professionals reporting actual use of AI tools (13). Comparing this figure to the study's own awareness rate of 89.1% highlights an even wider Saudi-specific awareness–use gap of approximately 57.5 percentage points in prior national evidence, underscoring that Saudi healthcare professionals’ favorable perceptions have historically outpaced actual clinical adoption by a wider margin than seen internationally. Additionally, earlier Saudi studies largely focused on general perceptions of AI, with limited examination of the underlying behavioral and organizational factors influencing adoption, and typically relied on small samples without a guiding theoretical framework. Organizational barriers most frequently implicated in the Saudi and regional literature include insufficient AI-specific training and continuing education, underdeveloped technical infrastructure and system interoperability, unclear data governance and regulatory guidance, and limited integration of AI tools into existing clinical workflows—barriers this study's facilitating-conditions and institutional-support findings (Sections 3.3–3.4) speak to directly. This situation highlights a critical knowledge gap in understanding not only whether healthcare professionals perceive AI positively but also why adoption remains limited and which factors facilitate or hinder its integration into daily clinical workflows. Despite this growing body of international evidence, limited empirical research has systematically examined these determinants in the Saudi healthcare context. This gap is particularly critical given Saudi Arabia's accelerated digital transformation under Vision 2030 and the substantial investments directed toward AI-enabled healthcare solutions. Without a clear understanding of healthcare professionals’ perceptions and contextual factors, AI initiatives may face resistance, inefficient implementation, and a limited impact on healthcare outcomes.

Therefore, this study aimed to (1) assess healthcare professionals’ attitudes toward AI, (2) examine perceptions of AI applications in healthcare practice, (3) identify factors influencing the adoption of AI technologies, and (4) evaluate healthcare professionals’ intentions and current use of AI tools in clinical practice based on the UTAUT model. This examination adds to the body of research by offering context-specific empirical evidence regarding healthcare professionals’ attitudes, perceptions, and factors affecting AI adoption in the Saudi healthcare system. Policymakers and healthcare leaders can utilize these findings to develop targeted strategies to promote sustainable AI adoption.

## 2 Materials and methods

### 2.1 Study design and participants

A nationwide cross-sectional online survey was conducted to investigate healthcare professionals’ attitudes, perceptions, and factors influencing the adoption of medical AI in Saudi Arabia. A quantitative approach was used to measure the study variables among healthcare professionals working across different healthcare settings, including hospitals, medical centers, primary care facilities, and administrative healthcare departments. The target population included both clinical professionals (physicians, nurses, and allied health professionals) and healthcare administrators (e.g., hospital managers and quality coordinators) working in governmental or private healthcare institutions across Saudi Arabia. The survey was distributed electronically, and responses were collected nationwide.

### 2.2 Inclusion and exclusion criteria

Participants were eligible if they were healthcare professionals currently residing and working in healthcare institutions in Saudi Arabia at the time of data collection. Both clinical and administrative healthcare professionals were included in the study. Students, interns, retired professionals, and individuals not currently practicing in a Saudi healthcare institution were not eligible to participate. Responses that were incomplete, duplicated, or did not meet the eligibility criteria were excluded prior to analysis. The final analytic sample consisted of 521 eligible healthcare professionals.

### 2.3 Sampling and data collection

In total, 521 participants completed the survey. Convenience sampling was used to recruit participants. The survey was conducted from March 10 to March 20, 2026. The questionnaire was distributed electronically using Google Forms through professional and social communication channels, including WhatsApp, LinkedIn, email distribution, and direct outreach to hospitals, medical centers, and primary healthcare facilities across Saudi Arabia. Because the survey was distributed through multiple open electronic channels using convenience sampling, the total number of healthcare professionals who received the survey invitation could not be determined. Therefore, the response rate could not be calculated.

Data were collected using a previously validated questionnaire developed by Dai et al. (8), grounded in the Unified Theory of Acceptance and Use of Technology (UTAUT), to evaluate factors influencing healthcare professionals’ adoption of medical AI. The instrument measures key constructs, including performance expectancy, effort expectancy, social influence, facilitating conditions, perceived risk, and behavioral intention to use AI technologies in clinical settings. The complete questionnaire is provided in Supplementary Material 1.

The questionnaire consisted of four sections: (1) demographic characteristics; (2) knowledge and perspectives about medical AI; (3) experiences and insights in using medical AI (if the participant had used medical AI); and (4) perceptions and intention to use medical AI. The demographic section included age (continuous), gender (male and female), region (Central, Eastern, Western, Northern, and Southern regions of Saudi Arabia), educational level (diploma, bachelor's, master's, and PhD), occupation (physicians, nurses, allied health professionals, and healthcare administrator/manager), hospital level (primary, secondary, and tertiary care hospitals), department, professional level (entry level, mid-level, and senior level), and years of experience (continuous). The second section assessed participants’ knowledge and attitudes toward AI, including awareness, prior exposure, and interest in AI applications. The third section examined experiences with AI use among healthcare professionals who had previously used AI in a clinical setting.

Finally, the fourth section assessed the factors of AI adoption based on the UTAUT, including performance expectancy, effort expectancy, social influence, facilitating conditions, perceived risk, and behavioral intention. The items in this section were measured using a five-point Likert scale ranging from 1 (strongly disagree) to 5 (strongly agree).

### 2.4 Ethical considerations

This study was conducted in accordance with the Declaration of Helsinki and was approved by the Institutional Review Board of King Saud University (Approval No. 26-0135). Informed consent was obtained from all participants prior to survey completion. Participation was voluntary, and all collected data were kept anonymous and confidential.

### 2.5 Statistical analysis

Data were analyzed using IBM SPSS Statistics version 27. First, descriptive statistics, including frequencies, percentages, means, and standard deviations, were used to summarize participants’ demographic characteristics, such as age, gender, profession, educational level, and years of experience. Categorical variables are presented as frequencies and percentages, while continuous variables are reported as means and standard deviations.

The primary dependent variable was intention to use medical AI. Independent variables were derived from the UTAUT model and included performance expectancy, effort expectancy, social influence, facilitating conditions, and perceived risk. The original validated instrument demonstrated high internal consistency across all UTAUT constructs, with Cronbach's alpha values exceeding 0.90 for performance expectancy, effort expectancy, social influence, facilitating conditions, perceived risk, and behavioral intention.

Pearson's chi-square test was used to examine differences in categorical variables across occupational groups. Second, multiple linear regression analysis was performed to ascertain the significant predictors of the intention to use medical AI; multiple linear regression was considered appropriate because the primary outcome variable (intention to use AI) was treated as a continuous variable derived from Likert-scale items. Finally, mediation analysis was conducted to explore potential indirect associations between facilitating conditions, performance expectancy, effort expectancy, and intention to use AI.

## 3 Results

### 3.1 Demographic characteristics of participants

This study included a diverse group of healthcare professionals, as shown in Table 1. Physicians were predominantly male (68.8%), whereas the nursing workforce was mostly female (57.3%). Most participants were located in the Central Region (62.8%), reflecting the region's large healthcare infrastructure. Educational level varied significantly, with postgraduate qualifications being most common among physicians (77.5%) and administrators (65.2%), compared to lower proportions among nurses and allied health professionals. Administrators also had the highest proportion of senior roles (73%) and the most work experience (over 32% had ≥16 years).

**Table 1:** Characteristics of the survey participants according to professional group (*N* = 521).

Characteristics	Total (*n* = 521)	Admin/Manager (*n* = 89)	Nurse (*n* = 75)	Physician (*n* = 160)	Allied Health (*n* = 197)
Gender: Female, *n* (%)	210 (40.3)	35 (39.3)	43 (57.3)	50 (31.3)	82 (41.6)
Gender: Male, *n* (%)	311 (59.7)	54 (60.7)	32 (42.7)	110 (68.8)	115 (58.4)
Region: Central, *n* (%)	327 (62.8)	60 (67.4)	33 (44.0)	107 (66.9)	127 (64.5)
Region: Eastern, *n* (%)	73 (14.0)	14 (15.7)	21 (28.0)	19 (11.9)	19 (9.6)
Region: Northern, *n* (%)	25 (4.8)	6 (6.7)	2 (2.7)	7 (4.4)	10 (5.1)
Region: Southern, *n* (%)	40 (7.7)	2 (2.2)	11 (14.7)	8 (5.0)	19 (9.6)
Region: Western, *n* (%)	56 (10.7)	7 (7.9)	8 (10.7)	19 (11.9)	22 (11.2)
Education: Bachelor's, *n* (%)	217 (41.7)	29 (32.6)	45 (60.0)	33 (20.6)	110 (55.8)
Education: Diploma, *n* (%)	16 (3.1)	2 (2.2)	6 (8.0)	3 (1.9)	5 (2.5)
Education: Postgraduate, *n* (%)	288 (55.3)	58 (65.2)	24 (32.0)	124 (77.5)	82 (41.6)
Hospital: Private, *n* (%)	170 (32.6)	32 (36.0)	19 (25.3)	37 (23.1)	82 (41.6)
Hospital: Public, *n* (%)	250 (48.0)	33 (37.1)	33 (44.0)	102 (63.7)	82 (41.6)
Hospital: Semi-gov, *n* (%)	101 (19.4)	24 (27.0)	23 (30.7)	21 (13.1)	33 (16.8)
Level: Primary, *n* (%)	184 (35.3)	24 (27.0)	30 (40.0)	46 (28.7)	84 (42.6)
Level: Secondary, *n* (%)	99 (19.0)	16 (18.0)	19 (25.3)	21 (13.1)	43 (21.8)
Level: Tertiary, *n* (%)	238 (45.7)	49 (55.1)	26 (34.7)	93 (58.1)	70 (35.5)
Title: Entry level, *n* (%)	51 (9.8)	3 (3.4)	12 (16.0)	19 (11.9)	17 (8.6)
Title: Mid-level, *n* (%)	150 (28.8)	21 (23.6)	26 (34.7)	32 (20.0)	71 (36.0)
Title: Senior level, *n* (%)	320 (61.4)	65 (73.0)	37 (49.3)	109 (68.1)	109 (55.3)
Experience: ≤ 5 y, *n* (%)	155 (29.8)	13 (14.6)	30 (40.0)	56 (35.0)	56 (28.4)
Experience: 6–10 y, *n* (%)	179 (34.4)	21 (23.6)	21 (28.0)	52 (32.5)	85 (43.1)
Experience: 11–15 y, *n* (%)	95 (18.2)	26 (29.2)	12 (16.0)	29 (18.1)	28 (14.2)
Experience: ≥16 y, *n* (%)	92 (17.7)	29 (32.6)	12 (16.0)	23 (14.4)	28 (14.2)

### 3.2 Knowledge and perspectives about AI

Awareness of medical AI was high across all groups (Table 2), especially among administrators (96.6%) and physicians (93.1%). However, actual use of AI showed sharper variation: physicians reported the highest usage (66.9%), while allied health professionals (38.6%) and nurses (40.0%) reported much lower levels. This distinction highlights unequal exposure to AI tools across professional roles.

**Table 2:** Awareness and previous use of medical AI by professional group (*N* = 521).

Characteristics	Total (*n* = 521)	Admin/Manager (*n* = 89)	Nurse (*n* = 75)	Physician (*n* = 160)	Allied Health (*n* = 197)	P value
Heard of medical AI? No	57 (10.9)	3 (3.4)	14 (18.7)	11 (6.9)	29 (14.7)	<.001
Heard of medical AI? Yes	464 (89.1)	86 (96.6)	61 (81.3)	149 (93.1)	168 (85.3)	
Ever used medical AI? No	254 (48.8)	35 (39.3)	45 (60.0)	53 (33.1)	121 (61.4)	<.001
Ever used medical AI? Yes	267 (51.2)	54 (60.7)	30 (40.0)	107 (66.9)	76 (38.6)	

At the sample level, the gap between having heard of medical AI (89.1%) and having ever used it (51.2%) was 37.9 percentage points, indicating that awareness has diffused across the workforce far faster than hands-on experience. This gap is uneven across roles: it is narrowest among physicians (93.1% aware vs. 66.9% used, 26.2 points) and administrators (96.6% vs. 60.7%, 35.9 points), and widest among allied health professionals (85.3% vs. 38.6%, 46.7 points) and nurses (81.3% vs. 40.0%, 41.3 points). This pattern suggests that awareness-building efforts have reached all groups fairly evenly, but access to hands-on AI tools—likely shaped by role-specific facilitating conditions, workflow integration, and institutional prioritization—has not kept pace, particularly for allied health staff and nurses.

### 3.3 Perceived institutional support and perspectives on AI

Among participants with prior AI experience, 61.8% felt that their institution showed a general to high interest in AI, although 38.2% perceived institutional attention to be low (Table 3). Optimism about the future of AI was strong across all professions, with physicians and nurses showing the highest levels (83% each). Importantly, optimism did not differ significantly across the groups, suggesting broad support for AI adoption.

**Table 3:** Perceptions of medical AI among healthcare professionals (*N* = 267).

Characteristics	Total (*n* = 267)	Admin/Manager (*n* = 54)	Nurse (*n* = 30)	Physician (*n* = 107)	Allied Health (*n* = 76)	*P* value
Hospital attention: general	99 (37.1)	22 (40.7)	10 (33.3)	37 (34.6)	30 (39.5)	.965
Hospital attention: high	66 (24.7)	13 (24.1)	9 (30.0)	26 (24.3)	18 (23.7)	
Hospital attention: low	102 (38.2)	19 (35.2)	11 (36.7)	44 (41.1)	28 (36.8)	
Prospects: optimistic	211 (79.0)	41 (75.9)	25 (83.3)	89 (83.2)	56 (73.7)	.381
Prospects: pessimistic	56 (21.0)	13 (24.1)	5 (16.7)	18 (16.8)	20 (26.3)	

The co-existence of high optimism (79.0%) with a substantial share reporting low institutional attention (38.2%) is not necessarily contradictory: these two items capture different constructs. Optimism reflects a personal belief about AI's long-term potential for the profession, largely independent of one's own workplace, whereas perceived institutional attention reflects a concrete judgment about the respondent's specific hospital's current level of investment and visible activity around AI. It is plausible—and consistent with the facilitating-conditions findings in Section 3.4, where only 33%–45% of respondents felt their institution provided adequate resources—that professionals believe in AI's promise in the abstract while simultaneously judging that their own institution has not yet operationalized that promise. This distinction reinforces that closing the intention–use gap is an organizational execution problem rather than a problem of professional buy-in.

### 3.4 Perceptions of and intention to Use medical AI

Participants generally reported positive perceptions toward the adoption of medical AI across all UTAUT domains, as shown in Table 4. The highest ratings were observed for **performance expectancy** (mean = 4.13, SD = 0.86), with approximately 75.2% of participants agreeing that AI could enhance clinical performance. Similarly, **effort expectancy** was rated highly (mean = 4.06, SD = 0.89), with 71.8% indicating confidence in their ability to learn and use AI tools. **Social influence** received comparatively lower ratings (mean = 3.24, SD = 1.02), with agreement ranging from approximately 33% to 46% among clinicians and reaching its highest level among healthcare administrators. **Facilitating conditions** also received relatively modest ratings (mean = 3.16, SD = 1.12), as only 37.0% of participants perceived that their organizations provided adequate resources and support for AI implementation. Participants reported moderate levels of **perceived risk** (mean = 3.29, SD = 0.92), with concerns focusing primarily on diagnostic errors and medicolegal responsibility rather than job replacement. Overall, **intention to use medical AI** was high (mean = 4.04, SD = 0.89), with 71.6% expressing willingness to adopt AI in practice. The highest intention to use was observed among healthcare administrators, followed by allied health professionals. Overall, these findings indicate strong individual readiness to adopt medical AI despite relatively limited perceptions of organizational support.

**Table 4:** UTAUT constructs and intention to use medical AI by professional group—construct-level summary.

Construct	Total Mean (SD)	Total Agree *n* (%)	Admin Mean (SD)	Nurse Mean (SD)	Physician Mean (SD)	Allied Mean (SD)
Performance expectancy	4.13 (0.86)	392 (75.2)	4.35 (0.73)	4.05 (0.91)	4.15 (0.88)	4.04 (0.86)
Effort expectancy	4.06 (0.89)	374 (71.8)	4.17 (0.83)	4.03 (0.91)	4.14 (0.94)	3.96 (0.89)
Social influence	3.24 (1.02)	188 (36.1)	3.52 (0.93)	3.28 (1.01)	3.20 (1.05)	3.15 (1.02)
Facilitating conditions	3.16 (1.12)	193 (37.0)	3.54 (1.10)	3.38 (1.04)	3.02 (1.22)	3.03 (1.12)
Perceived risk	3.29 (0.92)	194 (37.2)	3.46 (0.96)	3.27 (0.96)	3.24 (0.90)	3.26 (0.92)
Intention to use	4.04 (0.89)	373 (71.6)	4.39 (0.69)	4.01 (0.93)	3.98 (0.88)	3.93 (0.89)

Full item-level statistics for each construct are retained in the underlying dataset and available on request; the summary above presents the construct-level mean, standard deviation, and percentage agreement reported in the original analysis.

### 3.5 Factors influencing the intention to Use medical AI

Multiple linear regression analysis demonstrated that **performance expectancy** was the strongest predictor of healthcare professionals’ intention to use medical AI across all professional groups. The strongest association was observed among physicians (*B* = 0.565, *p* < .001), followed by allied health professionals, healthcare administrators, and nurses. **Effort expectancy** was the second strongest predictor, with the highest effect observed among nurses (*B* = 0.302, *p* = .015). **Facilitating conditions** were significantly associated with intention to use AI among nurses and healthcare administrators but were not significant among physicians or allied health professionals. In contrast, **social influence** was generally not associated with intention to use AI and showed a significant negative association only among nurses (*B* = –0.303, *p* = .001). **Perceived risk** was not significantly associated with intention to use AI in any professional group. Overall, these findings suggest that healthcare professionals’ intention to adopt medical AI is primarily driven by perceptions of its usefulness and ease of use rather than by external social influence or concerns about potential risks.

### 3.6 Mediating effects of performance expectancy and effort expectancy

**Figure 2:**
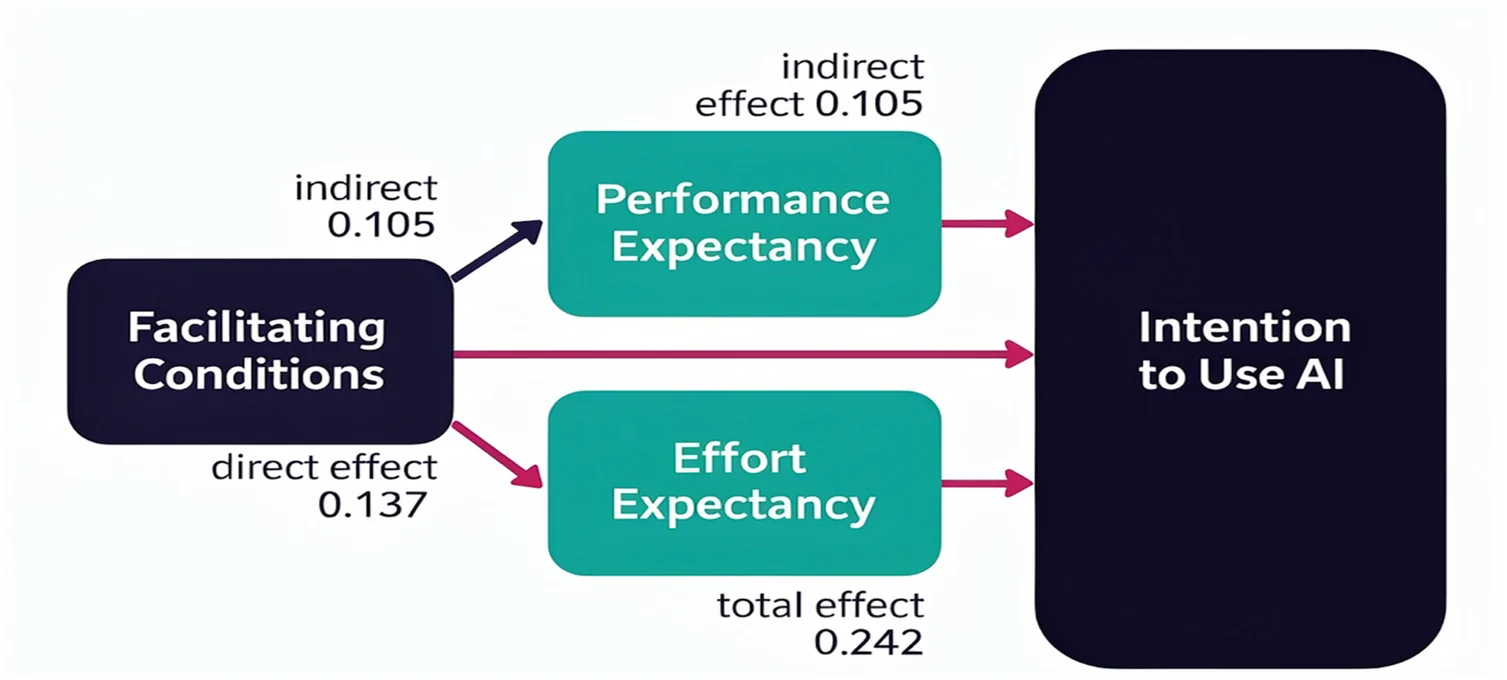
Conceptual mediation model showing the direct, indirect, and total effects of facilitating conditions on healthcare professionals’ intention to use artificial intelligence through performance expectancy and effort expectancy.

Institutional support influences intention mainly indirectly through performance and effort expectancy. Patterns consistent with indirect associations were observed across professional groups, as shown in Table 5. Among physicians, the association between facilitating conditions and intention to use AI appeared to operate primarily through performance expectancy and effort expectancy, with a fully indirect pattern (a near-zero, non-significant direct effect alongside a substantial indirect effect). A stronger indirect association was also observed among allied health professionals. These findings suggest that organizational support may be positively associated with AI adoption intentions by enhancing healthcare professionals’ perceptions of AI usefulness and ease of use. The mediating effects of performance expectancy and effort expectancy on the relationship between facilitating conditions and behavioral intention to adopt AI among healthcare professionals are illustrated in Figure 2.

**Table 5:** Mediation analysis results by professional group.

Effect	Total (*n* = 521)	Admin/Manager (*n* = 89)	Nurse (*n* = 75)	Physician (*n* = 160)	Allied Health (*n* = 197)
Total effect, *B* (95% CI)	0.242 (0.20–0.28)	0.284 (0.15–0.42)	0.341 (0.15–0.53)	0.182 (0.07–0.29)	0.238 (0.13–0.35)
Direct effect, *B* (95% CI)	0.137 (0.10–0.18)	0.211 (0.09–0.33)	0.247 (0.07–0.42)	−0.053 (−0.14 to 0.04)	0.053 (−0.05 to 0.16)
Indirect effect, *B* (95% CI)	0.105 (0.08–0.13)	0.073 (0.02–0.12)	0.094 (0.03–0.16)	0.235 (0.14–0.33)	0.185 (0.10–0.27)

## 4 Discussion

This study investigated Saudi healthcare professionals’ attitudes, perceptions, and intentions regarding the adoption of AI in clinical practice using the UTAUT. The findings reveal high optimism toward AI, consistent with global trends, yet practical adoption remains limited, demonstrating a persistent “intention–use gap.” Understanding why this gap exists provides a valuable direction for strengthening national AI strategies and guiding institutional decision-makers.

### 4.1 Attitudes toward AI

A strong positive attitude toward AI was clear, with 79.0% of participants expressing optimism about AI's role in future healthcare. This pattern aligns with international work, such as Dai et al. (8) in China and the American Medical Association and Center for Digital Health and AI (14) survey, both showing growing professional enthusiasm. Similarly, 85.7% of European primary care clinicians (15) reported awareness of AI's clinical potential. In the current study, awareness reached 89.1%, reflecting a particularly receptive professional environment. By contrast, findings from Egypt (11) revealed lower optimism (51.8%), suggesting that national digital progress plays a strong shaping role.

Saudi Arabia's comparatively higher awareness (89.1%) and optimism (79.0%) can be linked directly to this study's own institutional and demographic findings rather than to external factors alone. Awareness was highest among administrators (96.6%) and physicians (93.1%)—the two groups most exposed to Ministry of Health digital-transformation initiatives and to tertiary/large-hospital settings (Table 1 shows administrators and physicians concentrated in tertiary care, at 55.1% and 58.1% respectively). This concentration of awareness in leadership and physician roles, situated within a national policy push under Vision 2030 and coordinated by the Saudi Data and Artificial Intelligence Authority (SDAIA) and the Ministry of Health's eHealth programs, plausibly explains why Saudi awareness and optimism outpace the levels reported in less centrally coordinated health systems. High performance expectancy (4.13) and effort expectancy (4.06) demonstrate that healthcare professionals perceive AI as both beneficial and easy to adopt—core elements of the UTAUT model, and, per the regression results in Section 3.5, precisely the two beliefs most strongly associated with intention. The discovery has important implications for the implementation of Saudi Vision 2030 policies. Healthcare professionals want to embrace AI positively. However, optimism alone is insufficient; successful implementation requires national and organizational policies that tackle identified barriers through specific professional training, clear ethical and regulatory guidance, strong data governance, and sufficient technical and institutional support. For this positive orientation to translate into real-world adoption, national policy must ensure that enthusiasm is supported by practical infrastructure: clear competency standards, inclusion of AI skills in licensing frameworks, and clinical workflow designs that make AI intuitive rather than burdensome. When optimism is matched with supportive systems and training pathways, confidence becomes consistent adoption.

### 4.2 Perceptions of AI applications and the intention–Use Gap

Although awareness was high, only 51.2% had used AI tools, confirming a significant 37.9 percentage-point gap between awareness and actual implementation, and a 20.4 percentage-point gap between intention (71.6%) and actual use (51.2%). This mirrors findings from Hashem et al. (16) at the Alan Turing Institute, where only 25% of UK doctors reported AI use, and the Elsevier Clinician of the Future Survey (17), where only 16% used AI clinically.

This study's own findings, rather than external literature alone, explain much of the 20.4-point intention–use gap. Only 37.0% of participants reported adequate facilitating conditions (Section 3.4) and only 61.8% perceived general-to-high institutional attention to AI, meaning 38.2% perceived low institutional attention (Section 3.3). Because facilitating conditions and institutional support were both measured directly in this sample—and facilitating conditions emerged as a significant, if modest, direct predictor of intention among nurses and administrators (Table 6) the gap between the 71.6% who intend to use AI and the 51.2% who have actually done so is best explained as an organizational execution shortfall: professionals are ready, but roughly two-thirds of institutions have not yet supplied the resources, workflows, and access needed to convert intention into practice. Three contextual issues reinforce this interpretation:

**Table 6:** Multivariable linear regression analysis of factors associated with intention to use medical AI by professional group.

Predictor	Total B (95% CI); P	Physician B (95% CI); P	Nurse B (95% CI); P	Admin B (95% CI); P	Allied B (95% CI); P
Performance expectancy	0.491 (0.41–0.57); <.001	0.565 (0.42–0.70); <.001	0.366 (0.12–0.61); .004	0.488 (0.30–0.67); <.001	0.503 (0.37–0.63); <.001
Effort expectancy	0.224 (0.15–0.30); <.001	0.200 (0.07–0.32); .002	0.302 (0.06–0.55); .015	−0.071 (−0.26 to 0.12); .476	0.209 (0.08–0.34); .002
Social influence	0.014 (−0.05 to 0.08); .684	0.097 (−0.01–0.20); .082	−0.303 (−0.49 to −0.12); .001	0.049 (−0.10 to 0.20); .518	0.082 (−0.04 to 0.21); .202
Facilitating conditions	0.067 (0.01–0.12); .023	−0.053 (−0.14 to 0.04); .251	0.247 (0.07–0.42); .006	0.211 (0.09–0.33); .001	0.053 (−0.05 to 0.16); .328
Perceived risk	0.013 (−0.05 to 0.07); .672	0.051 (−0.05 to 0.16); .344	0.057 (−0.12 to 0.24); .537	−0.024 (−0.14 to 0.10); .698	0.002 (−0.10 to 0.11); .968

The regression models also adjusted for gender, education, hospital type, years of experience, and prior AI awareness/use; full coefficients for these covariates are retained in the dataset. Model fit statistics (*R*^2^, adjusted *R*^2^, *F*) are flagged above for author addition.

Limited facilitating conditions: Only 37.0% of participants reported adequate institutional support. This aligns with global evidence showing that insufficient training, high workloads, and weak organizational capacity hinder AI uptake (18, 19). Practically, this highlights the need for structured national training programs, hospital-level AI support units, and continuous technical mentorship, not merely purchasing technology.

Ethical and data privacy concerns: 37.2% expressed concerns consistent with the privacy and governance issues described by Ahmad et al. (20). Governance frameworks and standardized data protection protocols must become more visible and accessible to frontline workers, reducing hesitation and ambiguity.

Low institutional prioritization: 38.2% believed that their hospitals showed limited attention to AI, echoing Rogan et al. (21). This underlines the need for leadership engagement, without it, healthcare workforce motivation remains high, but direction remains unclear.

Despite these barriers, 71.6% of respondents expressed intention to use AI, showing that individual readiness is not the issue, organizational and system readiness is. Therefore, closing the intention–use gap requires a national policy that prioritizes workforce capability building, ethical clarity, and institutional commitment, rather than further efforts to persuade an already receptive workforce.

### 4.3 Factors influencing AI adoption

The regression results showed that performance expectancy (*B* = 0.491) and effort expectancy (*B* = 0.224) were the strongest predictors of intention. Professionals adopt AI when they believe it improves their performance and is easy to learn. Facilitating conditions were also modestly associated with intention, underscoring the role of organizational support. Interestingly, social influence had a limited impact overall and was even negative among nurses (*B* = − 0.303), and perceived risk was non-significant across every professional group.

The non-significant effect of perceived risk (*B* = 0.013, *p* = .672 overall) suggests that Saudi healthcare professionals’ intention to adopt AI is not meaningfully constrained by concerns about errors, medicolegal exposure, or automation, despite Section 3.4 showing moderate risk perception across groups (37.2% agreement). Practically, this indicates that risk-focused messaging (e.g., emphasizing safety guardrails) is unlikely to be the most efficient lever for increasing adoption; effort invested there may yield less than effort invested in demonstrating usefulness and usability. The negative and significant social-influence coefficient among nurses (*B* = − 0.303, *p* = .001) is a more consequential finding: unlike the other groups, nurses in this sample who perceived more social pressure to use AI reported lower intention, not higher. This pattern is echoed in European nursing settings, where hierarchical, protocol-driven environments sometimes discourage individual experimentation with new technology, and peer or supervisor pressure can be read as an imposed mandate rather than an invitation. As in Catalina et al. (15), perceived risk has no significant effect, confirming that the perceived value of AI outweighs concerns about automation or misdiagnosis. These findings collectively highlight that human-centered design and usability matter more than mandates or external pressures, particularly for nurses. Policies that simply “require” adoption are unlikely to be effective for this group specifically. Instead, developers should involve clinicians —nurses in particular—in design, testing, and customization, and workflow integration should prioritize practicality. When clinicians feel that AI tools are intuitive and aligned with their work, they will adopt them willingly rather than under pressure.

### 4.4 Unified theory of acceptance and Use of technology (UTAUT)

Consistent with global research, the current study found that performance expectancy and effort expectancy strongly predicted intention, whereas social influence and perceived risk were negligible. This pattern replicates prior UTAUT-based technology-adoption studies (e.g., Stoumpos et al. (22), confirming the model's core mechanism in a new national context. The study's distinct contribution is twofold: first, it shows that this UTAUT mechanism holds not only for physicians and nurses, the professions most UTAUT-based healthcare studies focus on—but also for administrators and allied health professionals, two groups rarely examined together in the same UTAUT model; second, the mediation analysis (Section 3.6) extends the standard UTAUT structure by showing that facilitating conditions operate largely indirectly, through performance and effort expectancy, rather than as an independent direct driver, a mechanism with clear practical implications, since it means that investing in facilitating conditions pays off specifically by changing how useful and how easy AI is perceived to be, not merely by providing resources in the abstract. In the Saudi context, this suggests that authorities should focus not on top-down enforcement but on shaping the two core perceptions that truly matter: usefulness and ease of use. This can be achieved through national demonstration projects in tertiary hospitals, AI competency frameworks, interprofessional workshops, and visible successful local-use cases. When early adopters are visible and supported, peer trust grows.

### 4.5 Mediating effects of UTAUT constructs

Mediation analysis showed that facilitating conditions influenced intention primarily indirectly through performance and effort expectancy. Among physicians, mediation was effectively complete (direct effect *B* = −0.053, non-significant; indirect effect *B* = 0.235), and among allied health workers, the indirect effect was also comparatively strong (*B* = 0.185) against a small, non-significant direct effect (*B* = 0.053). These two groups differ structurally from nurses and administrators, for whom facilitating conditions retained a significant direct effect on intention (Table 6) alongside a smaller indirect effect (Table 5). A plausible explanation is that physicians and allied health professionals, who typically interact with AI tools (e.g., imaging or laboratory decision-support systems) at arm's length from the institution's administrative resourcing decisions, experience facilitating conditions mainly by developing greater confidence that the tools work and are easy to use, rather than by directly weighing the resources themselves. Nurses and administrators, by contrast, are often closer to the operational deployment of resources (staffing, devices, protocols) and may translate facilitating conditions into intention more directly, without that belief needing to be re-routed through usefulness or ease-of-use perceptions first. This reinforces a key policy insight: resources alone do not cause adoption for every professional group equally, and for physicians and allied health staff specifically, users must experience genuine value before organizational investment translates into intention. Therefore, hospitals should create AI pilot programs in which clinicians can test systems in low-risk settings, receive live mentorship, and provide feedback. When infrastructure is paired with experiential learning, confidence increases, and adoption becomes self-sustaining, with targeted implementation strategies calibrated to how each professional group actually converts support into intention.

### 4.6 Strengths and limitations

This study has several limitations that should be considered when interpreting the findings. First, the cross-sectional design limits the ability to infer causal relationships between study variables; the observed associations between UTAUT constructs and intention to use AI are directional inferences drawn from theory, not evidence of causal effect, and reverse or reciprocal relationships (e.g., prior use shaping later perceived usefulness) cannot be ruled out. Second, participants were recruited through digital platforms (e.g., WhatsApp, LinkedIn, email, and professional networks) using convenience sampling, which may have introduced selection bias: healthcare professionals who were more digitally engaged were more likely to participate, while those with poor digital engagement, who may also hold different, possibly less favorable, attitudes toward AI, were likely under-represented. This means the true population-level awareness, optimism, and use rates in Saudi Arabia may be somewhat lower than reported here, and the findings should be generalized to the broader Saudi healthcare workforce with caution. Third, the study relied on self-reported responses for both AI use and UTAUT perceptions, which may be subject to recall bias and social desirability bias, potentially inflating reported awareness, optimism, and use relative to objectively verified behavior. In addition, because the survey was distributed through multiple open electronic channels using convenience sampling, the total number of healthcare professionals who received the survey invitation could not be determined. Consequently, the response rate could not be calculated, limiting the assessment of potential non-response bias. Despite these limitations, the study provides valuable nationwide insights into healthcare professionals’ perceptions and intentions regarding AI adoption in Saudi Arabia. Future research should consider longitudinal and mixed-method approaches to better examine changes in AI adoption over time, establish temporal precedence between UTAUT constructs and use, and explore potential causal relationships between organizational factors, perceptions, and AI adoption behaviors among healthcare professionals. Future research may also complement online distribution through social media and professional networks with offline recruitment methods, such as in-person recruitment at healthcare institutions and community-based sampling, to minimize the potential overrepresentation of digitally engaged individuals and improve representativeness.

## 5 Conclusion

This study's central and most policy-relevant contribution is the demonstration that Saudi healthcare professionals’ readiness for AI is not the bottleneck to adoption: awareness (89.1%), optimism (79.0%), and intention (71.6%) all substantially exceed actual clinical use (51.2%), and this gap is best explained by organizational rather than individual factors—modest facilitating conditions (37.0% adequate) and inconsistent institutional prioritization (38.2% perceiving low attention), operating largely through their effect on perceived usefulness and ease of use rather than as independent barriers. Saudi healthcare professionals show strong enthusiasm for AI, driven by beliefs about its usefulness and ease of use; however, actual adoption remains modest because of gaps in institutional support, training, and governance rather than resistance from professionals. This study confirms the relevance of the UTAUT framework in a previously underexamined national context spanning physicians, nurses, allied health professionals, and administrators, and highlights the importance of organizational support, workforce preparedness, and governance frameworks in facilitating AI adoption within healthcare settings.

Based on the significant predictors identified in the regression and mediation analyses, three evidence-based recommendations follow directly from the findings:

Strengthen institutional support and facilitating conditions specifically for allied health professionals and nurses—the two groups with the widest awareness–use gaps (46.7 and 41.3 percentage points, respectively)—through dedicated device/system access, workflow integration, and hospital-level AI support units, since facilitating conditions were a significant direct predictor of intention for exactly these groups.

Invest in AI-specific training and hands-on pilot programs that build performance expectancy and effort expectancy—the two strongest and most consistent predictors of intention across every professional group (*B* = 0.491 and 0.224 overall)—rather than campaigns aimed at persuading an already-optimistic workforce.

Establish clear national AI governance frameworks, ethical guidelines, and data-protection protocols at the institutional level to address the 37.2% of professionals expressing privacy/ethical concerns and to raise the 61.8% who currently perceive adequate institutional attention to AI toward system-wide coverage.

Strengthening these three areas may support the sustainable integration of AI into clinical practice in alignment with Saudi Vision 2030 healthcare transformation goals.

## Data Availability

The underlying data supporting the findings of this study are available from the corresponding author upon reasonable request.
