# Supplementary material for: Attitudes, perceptions, and factors influencing the adoption of artificial intelligence Among healthcare professionals in Saudi Arabia: a UTAUT-based study
Source: Front Health Serv. 2026 Aug 27;6:1895739. doi: 10.3389/frhs.2026.1895739 (PMC13558167; doi:10.3389/frhs.2026.1895739)
Supplement: Supplementary file 1 [file Supplementaryfile1.pdf]

Supplementary material 1.
Appendix: Study Questionaire

Consent form:

Dear Participant,
The purpose of this survey is to assess healthcare professionals' attitudes and perceptions toward artificial intelligence (AI) in healthcare and to identify the factors influencing its adoption**.**
This study is conducted **for academic research purposes. We** assure you that all the information you provide will remain strictly **confidential**. Your responses will be stored anonymously. Your participation is **completely voluntary**, and you may withdraw at any time without any obligation.
I consent to participate in this study and agree to the publication of the data generated from it:
☐ Yes ☐ No

Part 1: Demographic Characteristics
1. What is your gender?☐ Male
☐ Female

2. What is the province where you reside?
☐ Central Region
☐ Eastern Region
☐ Western Region
☐ Southern Region
☐ Northern Region

3. What is your highest degree of education?
☐ Diploma
☐ bachelor's degree
☐ Postgraduate degree (Master's/PhD)

4. What is your occupation?
☐ Physician
☐ Nurse
☐ Radiology, Laboratory, Rehabilitation, or other allied health professional (please specify): ___
☐ Healthcare Administrator / Manager

5. What type of hospital do you work in?
☐ Public hospital

☐ Private hospital
☐Semi-governmental hospital

6. What is the level of the hospital where you work?
☐ Primary hospital
☐ Secondary hospital
☐ Tertiary hospital7. What is your professional title level?
☐ Entry level
☐ Mid level
☐ Senior level

8. How many years have you been working?
☐ ≤5 years
☐ 6–10 years
☐ 11–15 years
☐ ≥16 years

Part 2: Knowledge and Perspectives about Medical AI
1. Have you ever heard of medical AI?
☐ Yes
☐ No

2. Have you ever used medical AI?
☐ Yes
☐ No

3. What is the level of attention your hospital pays to medical AI?
☐ Low attention
☐ General attention
☐ High attention

4. What is your view on the prospects of medical AI?
☐ Pessimistic view
☐ Optimistic view

5. In which areas of application are you more interested in medical AI?
(Multiple choices allowed)
☐ Assistive diagnosis
☐ Personalized treatment
☐ Clinical decision support☐ Patient monitoring

☐ Disease surveillance
☐ Medical robotics
☐ Medical data management
☐ Telemedicine
☐ Teaching and research
☐ Administrative use

Part 3: Experiences and Insights in Using Medical AI
(If you have used medical AI, please continue to answer. If not, please skip this part.)

1. When did you start using medical AI?
☐ ＜6 months
☐ 0.5–1 years
☐ 1–2 years
☐ 2–3 years
☐ ＞3 years

2. How satisfied are you with medical AI?
☐ Very dissatisfied
☐ Dissatisfied
☐ Neutral
☐ Satisfied
☐ Very satisfied

3. What is the main application for which you use medical AI? (Multiple choices allowed)
☐ Assistive diagnosis
☐ Personalized treatment
☐ Clinical decision support
☐ Patient monitoring
☐ Disease surveillance
☐ Medical robotics
☐ Medical data management
☐ Telemedicine☐ Teaching and research
☐ Others: _____________________

4. What aspects do you think need to be improved in the use of medical AI?
(Multiple choices allowed)
☐ Improve usability
☐ Enhance system stability and reliability
☐ Increase system response speed and processing power

☐ Improve cost-effectiveness
☐ Strengthen data security and privacy protection
☐ Enhance patient interaction features
☐ Offer more personalized service options
☐ Strengthen integration with existing medical workflows
☐ Provide more training and technical support
☐ Others: ____________________

Part 4: Perceptions and Intention to Use Medical AI
(Adoption of Likert 5-level scale from "Strongly disagree" to "Strongly agree," assigning values from 1 to 5.)
Response options for all items:
☐ Strongly disagree ☐ Disagree ☐ Neutral ☐ Agree ☐ Strongly agree

1. I think the application of medical AI can enhance my professional skills.
2. I think the application of medical AI can improve my work efficiency.
3. I think the application of medical AI can alleviate my work-related stress.
4. I think the application of medical AI can improve the healthcare experience and enhance
5. patient satisfaction.
6. I think I can easily master the use of medical AI.
7. I think through institutional training, organizational guidance, or assistance from others, I
8. can master the use of medical AI.7. My colleagues or peers are using medical AI.
9. My organization, colleagues, or peers recommend that I use medical AI.
10. Patients themselves hope to utilize medical AI services or products.
11. My organization can provide the necessary funds, equipment, products, or other resources required for using medical AI.
12. My organization can provide the technical support, training, or guidance needed for using medical AI.

13. When issues arise with medical AI products, developers or the organization can assist me in resolving them promptly.

14. I am concerned that medical AI may provide incorrect information, leading to adverse outcomes.

15. I am concerned that medical AI may result in medical disputes, making it difficult to determine responsibility.

16. I am concerned that the lack of transparency and explainability in medical AI may negatively impact the medical process.

17. I am concerned that the use of medical AI may exacerbate inequities in healthcare services.

18. I am concerned that my job might eventually be replaced by medical AI.

19. I am willing to use medical AI to assist me in my work.

20. I am willing to recommend that my colleagues or peers use medical AI to assist in their work.

21. I am willing to recommend that patients use medical AI products or services.

22. I am willing to engage in continuous learning, update my knowledge, and stay informed about the latest developments and technological advancements in the field of medical AI.
